# Depression among Online Respondent Oral Healthcare Workers during COVID-19 Pandemic: A Descriptive Cross-sectional Study

**DOI:** 10.31729/jnma.6421

**Published:** 2022-07-31

**Authors:** Saroj Prasad Deo, Anoop Krishna Gupta, Tarakant Bhagat, Harendra Mohan Singh

**Affiliations:** 1 Department of Oral and Maxillofacial Surgery, National Medical College, Birgunj, Parsa, Nepal; 2 Department of Psychiatry, National Medical College, Birgunj, Parsa, Nepal; 3 Department of Public Health Dentistry, B.P. Koirala Institute of Health Sciences, Dharan, Sunsari, Nepal; 4 Department of Conservative Dentistry and Endodontics, National Medical College, Birgunj, Parsa, Nepal

**Keywords:** *COVID-19*, *depression*, *oral health*

## Abstract

**Introduction:**

COVID-19 outbreak brought unprecedented pressure on dental and oral health care workers leading to increased depression. This study aimed to find the prevalence of depression among online respondent oral healthcare workers during the COVID-19 pandemic.

**Methods:**

A descriptive cross-sectional study was conducted from 24 June 2020 to 13 July 2020 among oral health care workers in a tertiary care centre. Ethical approval was taken from the Ethical Review Board (Reference number: 2710). Convenience sampling method was used. The data were collected using a questionnaire through Google Forms. Point estimate and 95% Confidence Interval were calculated.

**Results:**

Among 133 oral health care workers, the prevalence of depression was found to be 29 (21.80%) (14.78-28.82, 95% Confidence Interval).

**Conclusions:**

The prevalence of depression among oral health care workers was lower than similar studies done in similar settings.

## INTRODUCTION

Coronavirus Disease-19 (COVID-19) is described as the worst public health crisis in a generation, particularly for health care workers (HCWs).^1^ Depression is most common, urgent, yet to be appreciated among oral HCW, although it has been most frequently described in the literature. Its prevalence among HCWs ranges from 21.53% to 32.77% in developed nations, much lower than that of developing countries up to 31 to 37.5%.^2,3^ Moreover, its prevalence is highest (60%) among oral HCWs.^4,5^

According to the World health organisation (WHO), depression has been mentioned as the most common behavioural disorder, leading to distress, boredom, passivity, worthlessness, insomnia, and decreased concentration.^6^ Depressed oral HCWs lose their confidence and feel embarrassed and stressed at work, resulting in poor performance and low productivity in the workplace. Under this embarrassing condition, the ability of oral HCWs to cope with stressful situations declines and the incidence of professional negligence increases dramatically.

This study aimed to find out the prevalence of depression among online respondent oral healthcare workers during COVID-19 pandemic.

## METHODS

A descriptive cross-sectional study was conducted via an online web survey among oral health care workers (HCWs) during the COVID-19 pandemic. Data collection took place from 24 June 2020 to 13 July 2020 after taking ethical approval from the Ethical Review Board of the Nepal Health Research Council (Reference number: 2710). The participants were oral HCWs which included junior residents, senior residents, faculty members, medical officers/dental surgeons, dental nurses, dental hygienists, dental chairside assistants, technicians, clerical staff, administrators, security staff, sanitation workers, and maintenance worker drivers. Duplicated entries, incompletely filled forms, or those who wanted to drop out of the study within two weeks of submission were excluded from the study. Convenience sampling method was used.

Sample size was calculated by using the following formula:


$$\begin{array}{ll} \text{n} & ={\text{Z}}^{2}\times \frac{\text{p}\times \text{q}}{{\text{e}}^{\text{2}}}\\ & =1.96^{2}\times \frac{0.50\times 0.50}{0.10^{2}}\\ & =97 \end{array}$$

Where,

n = minimum required sample size
Z = 1.96 at 95% Confidence Interval (CI)
p = prevalence taken as 50% for maximum sample size calculation
q = 1-p
e = margin of error, 10%

The minimum required sample size was 97. In the calculated sample size, 10% was added to address the non-response rate after which the required sample size was 107. However, a sample size of 133 was taken for the study.

Mental health issues were assessed using the well-established 21-item Depression, Anxiety and Stress Scale (DASS-21). DASS-21 is a set of three selfreported scales designed to measure depression, anxiety and stress. Each component DASS category contained seven items scored on a 4-point Likert scale ranging from 0 (did not apply to me at all) to 3 (applied to me very much, or most of the time). Scores for depression was calculated.^7^

An informed e-consent was obtained from individual participants before enrollment in this study. The questionnaire was sent as Google Forms to oral HCWs via different social media platforms. The participants were recruited by sending the survey link to dental and oral HCWs through different electronic platforms. The data were extracted to Microsoft Excel 2016 from the Google Form. Data analysis was performed using IBM SPSS Statistics 11.5. Point estimate and 95% CI were calculated.

## RESULTS

Among 133 oral HCWs during the COVID-19 pandemic, the prevalence of depression was found to be 29 (21.80%) (14.78-28.82, 95% CI) (Table 1).

**Table 1:** The severity of depression according to the DASS depression score (n= 29).

Depression	n (%)
Mild	9 (31.03)
Moderate	13 (44.82)
Severe	5 (17.24)
Extremely severe	2 (17.24)

Of the 29 oral HCWs with depression, the majority 17 (58.62%) were males, 13 (44.82%) were single, 27 (93.10%) did not have any comorbidities and 14 (48.27%) were dental surgeons. The mean age was 31±7.42 years (Table 2).

**Table 2:** Demographic characteristics of oral healthcare workers with depression (n= 29).

Variables	n (%)
**Age (years)**	
20-29	12 (41.37)
30-39	13 (44.82)
>40	4 (13.79)
**Sex**	
Male	17 (58.62)
Female	12 (41.37)
**Marital status**	
Single	13 (44.82)
Married and living with spouse	15 (51.72)
Married and staying away from the spouse	1 (3.44)
Widow	-
Morbidity	2 (6.89)
Designation	
Junior resident	6 (20.68)
Senior resident	-
Faculty member	9 (31.03)
Dental surgeon	14 (48.27)
Sanitation worker	-
Place of current work	
Clinically active during outbreak	13 (44.83)
Halt clinical work	14 (48.27)
Involved in academic activity only	2 (6.89)
**Type of institution**	
University/Medical/Dental teaching college	16 (55.17)
Private dental hospital	7 (24.13)
Government hospital	6 (20.68)

## DISCUSSION

In an attempt to understand the COVID-19, this study examinedturmoil effect of depression amonge oral health workers in Nepal during the early phase of the COVID-19 pandemic. Although it was rampant among other health care workers, it was not much assessed among dental professionals in the past. This study could probably be next to comprehensive studies conducted.^8-12^ The prevalence of depression was 21.81% among oral HCWs. This study's results were significantly lower than those obtained in a recent study of HCWs in Nepal during the early stages of the COVID-19 pandemic.^13,14^ According to the research, 37.5 percent of Nepalese HCWs have depression symptoms.^14^ This could be due to the demanding nature of clinical work with no knowledge about the disease among health care workers. Sometimes, they may fear knowing information about exposure risks and COVID-19 infection.

The highest depression was found among oral HCWs in studies during the early pandemic. The prevalence of depression was 60% was reported among dental professionals.^9,11^ The main factors of the generation of depression among oral HCWs have been reported as social isolation, inability to meet with friends, concern about personal health, fear of someone in the family becoming infected, and lack of certainty about the future.^6,8^ Compared with those studies, our study reported significantly lower levels of depression. This could have occurred due to psychological maladaptation, mental fatigue, burnout, or early mitigation of psychological issues in our country.^6,7^ Similarly, another study found the risk of elevated psychological distress in 11.5% of the participants.^9^

In our study, the causes of the generation of psychological symptoms are mainly due to three concerns; first, immediate health impacts of the virus. Then there's the physical isolation's repercussions. Third, it's because of socioeconomic limitations. In Nepal, the national lockdown was started on 24 March 2020; most dental services were shut down until 1 July 2020. The lockdown involved significant restrictions on citizens' way of life, including measures such as 'staying at home, social distancing and the closure of workplaces, shops and other services. Such strict measures were continued on and off over a long period.^13,14^

On the intragroup comparison, this study found higher depression in males than in females. The past evidence suggests a wide gender gap for psychological issues among HCWs.^15^ In the age group categories, 3040 years exhibited the highest psychological issues; probably, they have high self-awareness and concern for their increased age and family health.^11^ Moreover, these age groups are critical active working forces in society and are, therefore, affected mainly by redundancies and dental clinic closures.^16^ Interestingly, we found more significant levels of depression among faculties at higher occupational posts.

Similarly, previous studies found an association between education levels and anxiety and depression levels.^6,17^ A high prevalence of depression could be due to higher levels of education resulting in higher knowledge, awareness and practices related to COVID-19 infection. Surprisingly, one of the studies found that lower occupation posts (nursing staff) exhibited a higher prevalence of anxiety and depression than faculties.^18^ Those results may be partly confounded because nurses are primarily female with higher psychological issues than males. Nurses face a greater risk of exposure to COVID-19 patients as they spend more time and provide direct care to patients. Likewise, dental and oral HCWs are vulnerable groups with a high exposure risk to COVID-19 patients as they work in close physical contact with patients (face to face) and have a longer duration of work.^19^ Unfortunately, our study did not find the above correlation, although most working clinicians were young and had faced a greater exposure risk to COVID-19 patients during the procedure. The finding could have occurred because many of the study respondents halted their clinical activity during the lockdown. They were probably unaware of the severity of the COVID-19 pandemic. Eventually, fewer psychological issues were found in dental surgeons. There is enough scientific evidence for factors associated with the generation of depression during the COVID-19 outbreak. These are predictable shortages of supplies (lack of logistics, deprivation of protective gears, food and transport).^20-25^

This study emphasizes the importance of oral HCWs in oral health care and emergency surgery management in society. Any changes that concern oral HCWs regarding their mental health difficulties should be addressed as soon as possible, and they should seek professional and family help. Workshops on mental health could be conducted to increase self-efficacy and widen the dentistry curriculum.

Small sample size and use of convenience sampling could limit the generalisability for all oral HCWs. The small sample size could be explained by high non response rate than expected due to short period of data collection. The study was of only 3 weeks period and didn't include long-term follow-up for the non respondents. Furthermore, this study was unable to identify factors linked to the cause of depression.

## CONCLUSIONS

The prevalence of depression among oral health care workers was lower than the similar studies done in similar settings. However, this prevalence of depression in oral health care workers emphasises the need for psychological intervention during health crisis situations.
